# Insights into Mechanisms of Tumorigenesis in Neuroendocrine Neoplasms

**DOI:** 10.3390/ijms221910328

**Published:** 2021-09-25

**Authors:** Lorenza Pastorino, Federica Grillo, Manuela Albertelli, Paola Ghiorzo, William Bruno

**Affiliations:** 1 Genetics of Rare Cancers, IRCCS Ospedale Policlinico San Martino, Largo Rosanna Benzi 10, 16132 Genoa, Italy; lorenza.pastorino@unige.it (L.P.); paola.ghiorzo@unige.it (P.G.); 2 Department of Internal Medicine and Medical Specialties (DiMI), University of Genoa, V.le Benedetto XV 6, 16132 Genoa, Italy; manuela.albertelli@unige.it; 3 Anatomic Pathology Unit, IRCCS Ospedale Policlinico San Martino, Largo Rosanna Benzi 10, 16132 Genoa, Italy; federica.grillo@unige.it; 4 Anatomic Pathology Unit, Department of Surgical Sciences and Integrated Diagnostics (DISC), University of Genoa, 1632 Genoa, Italy; 5 Endocrinology Unit, IRCCS Ospedale Policlinico San Martino, Largo Rosanna Benzi 10, 16132 Genoa, Italy

**Keywords:** neuroendocrine tumors, tumorigenesis, genomic integrity, chromatin stability, splicing, animal models

## Abstract

Genomic studies have identified some of the most relevant genetic players in Neuroendocrine Neoplasm (NEN) tumorigenesis. However, we are still far from being able to draw a model that encompasses their heterogeneity, elucidates the different biological effects consequent to the identified molecular events, or incorporates extensive knowledge of molecular biomarkers and therapeutic targets. Here, we reviewed recent insights in NEN tumorigenesis from selected basic research studies on animal models, highlighting novel players in the intergenic cooperation and peculiar mechanisms including splicing dysregulation, chromatin stability, or cell dedifferentiation. Furthermore, models of tumorigenesis based on composite interactions other than a linear progression of events are proposed, exemplified by the involvement in NEN tumorigenesis of genes regulating complex functions, such as *MEN1* or *DAXX*. Although limited by interspecies differences, animal models have proved helpful for the more in-depth study of every facet of tumorigenesis, showing that the identification of driver mutations is only one of the many necessary steps and that other mechanisms are worth investigating.

## 1. Introduction

Neuroendocrine neoplasms (NENs) are a group of neoplasia that arise from endocrine organs, e.g., pituitary, thymus, and adrenal gland medulla, and from the cells of the diffuse neuroendocrine system, e.g., lung, pancreas, gastrointestinal tract, paraganglia, and C-cells of the thyroid.

The clinical heterogeneity of NENs, in terms of site, appearance, and biological behavior, echoes the complexity of genetic and epigenetic bases of their tumorigenesis, with significant differences constantly discovered among various types of NENs, making it increasingly difficult to build a common model [1,2].

The annual incidence of NENs has risen in the last decade up to more than 3 per 100,000 people depending on the site of origin [3,4]. The possible reasons for this steady rise are represented by the availability of better diagnostic tools and a true rise in incidence. Overall survival has also shown a steady improvement, probably due to earlier diagnosis and better treatment modalities.

The classification of NENs has been refined over the years. Indeed, from an essentially descriptive but not prognostically relevant classification in the 1980s to 1990s, the introduction of differentiations, i.e., well-differentiated and poorly differentiated neoplasms, and, more recently, grade (mitotic index and proliferation index based on Ki67), have permitted the prognostic stratification of NENs. In particular, well-differentiated neuroendocrine tumors (NETs) show an organoid morphology with bland cytology, and proliferation index stratifies tumors into G1 (mitotic index <2 mitoses/10 high-power fields and/or Ki67 <3%), G2 (mitotic index between 2 and 20 mitoses/10 high-power fields and/or Ki67 between 3 and 20%), and G3 (mitotic index >20 mitoses/10 high-power fields and/or KI67 >20%). On the other hand, poorly differentiated neuroendocrine carcinomas (NECs) show atypia, solid growth patterns with either small or large cells with necrosis and high proliferation indexes (G3). This classification system applies to all gastroenteropancreatic NENs while lung NENs still use a classification specific for the thoracic site [5,6,7]: typical (low-grade) and atypical (intermediate-grade) carcinoids and high-grade large-cell neuroendocrine carcinomas and small-cell lung cancer (SCLC). The differential diagnosis of site of origin in metastatic, well-differentiated NETs may be possible using a variety of immunohistochemical markers, while these are rarely helpful in identifying origin in poorly differentiated NECs’ metastases [8]. Briefly, immunomarkers for intestinal (CDX2), pancreatic (PDX1 and ISL1), and lung (TTF1 and OTP) origins can be integrated in immunomarker algorithms that help in defining site of origin [9,10].

Though the histologic classification systems are prognostically relevant and are the basis for treatment choice, the identification of a clinically useful molecular classification is still lacking. Notwithstanding this, evidence on mechanisms of tumorigenesis have increased considerably in recent years due to genomic studies, which have identified a group of genetic pathways largely involved in the most frequent types of NENs.

However, further findings have highlighted that some pieces of the pathogenesis puzzle are still missing.

It is well established that up to 20% of NETs represent a clinical sign of one of at least 10 cancer-prone syndromes associated with high or moderate risk for their development, e.g., Multiple Endocrine Neoplasias (MEN) type 1, 2A, 2B, and 4, Von Hippel-Lindau syndrome, Tuberous Sclerosis complex, Familial Isolated Pituitary Adenoma, Pheochromocytoma/Paraganglioma syndromes, Hyperparathyroidism/Jaw tumor syndrome, and Neurofibromatosis type 1 [11].

In the context of these syndromes, the upstream genetic cause is clearly identified and valuable for the personalization of surveillance protocols or the identification in a family of individuals at risk. However, the consequential events directly related to a susceptibility germline variant are still incompletely described.

In addition, the vast majority of NETs are apparently sporadic. However, more than 10% of a European cohort appeared to be related to pathogenic germline variants in DNA damage repair (DDR) genes *MUTYH*, *CHEK2,* and *BRCA2*, the first known to be correlated to MYH-associated polyposis and the others to a higher risk for breast cancer [12].

Understanding the genetic and epigenetic interactions underlying the development of NENs is ever more relevant due to the paucity of viable biomarkers and the potential translation of these data in clinical practice.

This review aimed to merge recent insights on NENs’ tumorigenesis, integrating up-to-date reviews [13,14], collecting results from various basic research studies, highlighting peculiar and more in-depth mechanisms that pave the way for further studies, and showing paradigms of tumorigenesis that could be worth investigating even for other kinds of tumors.

To date, the genes described as shared among different types of NENs, and with a significant role in their tumorigenesis, are characterized by having complex functions and interactions and are involved in chromatin remodeling and telomere maintenance (*DAXX, ATRX*), cell proliferation (*CDKN1B*), DDR (*MGMT*), and mTOR pathway (*PTEN, TSC1/2*), not to mention the most important genetic player, *MEN1*. *MEN1* encodes a ubiquitous scaffold protein coordinating dozens of proteins whose functions range from chromatin and transcriptional status to miRNA biogenesis (as explored in other recent reviews [15,16]).

Indeed, the review illustrated recent or preliminary evidence, in particular from mouse and zebrafish models or cell studies, that strengthen previous and well-established results concerning the aforementioned as well as other genes, unraveling further aspects of their role in NENs’ tumorigenesis, but also identifying consistent novel players beyond the genetic data with wide-ranging effects on a transcriptional level. Overall, this highlighted that comprehension of NENs’ tumorigenesis is far from being exhausted.

**Figure 1:**
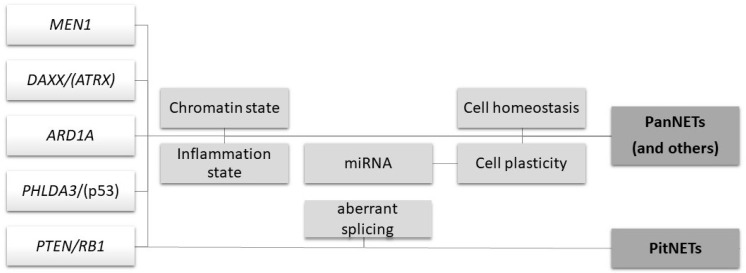
An overview of the main mechanisms involved in NENs’ tumorigenesis. This intersectional picture summarizes the tumorigenesis mechanisms described in the review. The first white part includes the main genes involved. The central light-gray part includes the consequences of the alterations of the involved genes or other intertwined players, e.g., miRNA or aberrant splicing (for PitNETs). The third dark-gray part includes the NENs as the final results of these processes.

Considering this complexity, this review aimed to integrate the consolidated multistep models starting from the description of novel tumor suppressor genes, to move towards a picture of intergenic cooperation and other upper layers of the gene expression alteration. Examples of the latter include the splicing dysregulation or the modification of chromatin status, not too unsurprising due to the functions of the most frequently involved genes, but also seemingly unique players, such as endogenous retroviral sequences or specific miRNA clusters with a main role in the process of cell dedifferentiation (see Table 1 and Figure 1 for a summary).

## 2. Novel Tumor Suppressor Genes (TSGs)

Recent whole-genome sequencing studies [13,17,18] show that p53 is rarely altered in NETs, e.g., low-grade lung NETs, pancreatic NETs (PanNETs), and gastro-intestinal NETs, despite its known frequent alteration in many cancers.

Nevertheless, p53 functions through various downstream substrates, such as *PHLDA3* (Pleckstrin Homology Like Domain Family A Member 3), a TSG which inhibits, in contrast to its activation by PI3K/PIP3 signaling, the oncoprotein Akt [26,27], also negatively regulated by p53 through *PTEN* (Phosphatase and TENsin homolog), a gene altered in up to 26.4% of human PanNETs [13,28] and reported as downregulated in Pituitary NETs (PitNETs) [29,30].

Any imbalance on p53-Akt opposite regulation may have a central role in tumorigenesis. It has been demonstrated that 91% of lung NETs and 72% of PanNETs have a functional loss or LOH of either p53 or *PHLDA3,* respectively [26,31].

In addition, loss of *PHLDA3* in MIN6 cells (mouse pancreatic islet cells), was shown to lead to activation of PI3K/Akt/mTOR pathway and hyperplasia, as also demonstrated by the administration in *PHLDA3*-deficient mice of streptozotocin, inducing apoptosis in pancreatic islet cells, which ultimately resulted as resistant but without tumor appearance [26].

These results impact therapy protocols emphasizing that, instead of administering streptozotocin to patients affected by PanNETs [32], PI3K/Akt/mTOR pathway inhibitors, e.g., everolimus [33], are also of choice in the presence of *PHLDA3* loss.

Furthermore, molecules simulating PHLDA3-mediated inhibition could also be efficient in other cancers presenting Akt activation [34].

Interestingly, in PanNETs, a two-hit loss of *PHLDA3* is comparable, and in cooperation with PanNETs’ progression, to that of *MEN1*, whose mutations are known to be highly frequent (up to 60%) in PanNETs [12,19]. Indeed, an association with a worse prognosis, as opposed to *MEN1* loss alone, was shown, defining *PHLDA3* loss as a novel malignancy potential biomarker. The same combination was observed in rectal NETs.

In summary, it will be worth investigating if *PHLDA3*, found to be functionally deficient in PanNETs, lung NETs, and rectal NETs, is a TSG for even other subtypes of NETs or wild-type p53 cancers, also in the perspective of targeted therapies.

## 3. Cooperative Tumorigenic Effects

The model presented in the previous paragraph consolidates the hypothesis of combined tumorigenic effects, as explored in other studies.

A paper from Xu et al. [20] focused on the reciprocally reinforcing interactions between p53 and *RB1* or *MEN1* and *PTEN* with tissue-specific effects in mice with homozygous deletion of TSGs.

For example, the study recalled p53 is altered in only 4% of PanNETs but it has been described in association with *RB1* alterations in the poorly differentiated PanNECs [35], and about 90% of PitNETs have at least one *RB1* pathway gene silenced due to promoter methylation [36,37].

While the deletion of the p53 gene alone is insufficient to initiate NET development, *RB1* mouse models developed PitNETs [38], and p53 deletion accelerated their development [39].

Compound mice with *MEN1* and *PTEN* deletions developed PitNETs and PanNETs [40,41,42], while *p18−*/−*Pten*+/− mice developed PitNETs [43,44], suggesting that *PTEN* plays a role in pancreatic islet and also pituitary tumorigenesis.

The authors performed single and pairwise deletion of the above TSGs in pituitary and pancreatic islets using the Cre-LoxP system [45,46], rating their role in NETs’ development. In particular, in PitNETs the order of relevance in initiation and/or progression was established as *RB1*, *PTEN*, *MEN1,* and p53 while, as expected, in islet tumorigenesis it was *MEN1*, *PTEN*, *RB1,* and lastly p53.

A study on NECs, such as SCLC, highlighted the loss of both p53 and *RB1* in about 80% of cases, a combined late event that drives lineage plasticity, e.g., as a mechanism of resistance to treatment [47,48].

This is a further example of a multistep tumorigenesis model, which, though consistently described, has yet to be fully explored with regards to transcriptional profiling necessary to disclose any gene involved, tissue specificity, and the stage of the tumor development in which it is relevant.

## 4. Dysregulation of Splicing Machinery in PitNETs

Genome-Wide Association Studies (GWAS) and mouse model studies revealed some of the genes that play a major role in NET tumorigenesis. Nevertheless, the relevance of driver mutations varies from gene to gene and for the different types of NETs. The studies described above also highlighted the role of some TSGs in PitNETS, known to be mostly sporadic, though somatic or shared mutations have been described.

Beyond genetic somatic events, subcellular molecules such as cytoskeleton and scaffold proteins, involved in somatostatin and dopamine receptors’ functionality, have been described as relevant players in the biological aggressiveness or therapeutic resistance of PitNETs, as well as transcriptomic alterations and miRNAs [49], though this evidence is too preliminary for therapeutic implementation.

Furthermore, clinical and therapy resistance biomarkers for NETs are far from being standardized and routinely adopted in clinical practice, but this is particularly accurate for a subgroup of PitNETs which, while mostly benign, can have an aggressive course, e.g., those underpinned by a germline predisposition determining syndromic conditions (*MEN1, CDKN1B*) or familial isolated pituitary adenomas (*AIP*).

Interestingly, while syndromic conditions are associated with genes involved in cell-cycle and/or transcription, isolated pituitary adenomas and sporadic cases are mainly correlated with genes involved in calcium and cAMP signaling [50], such as the aforementioned *AIP* (Aryl Hydrocarbon Receptor Interacting Protein), *GPR101* (G Protein-Coupled Receptor 101), *PRKAR1A* (Protein Kinase CAMP-Dependent Type I Regulatory Subunit Alpha), or *GNAS* (α subunit of the stimulatory G protein) specifically in growth hormone (GH) PitNETs.

Indeed, most cases of PitNETS are sporadic and associated with somatic genetic variants not correlated with a more aggressive phenotype, e.g., also in *USP8* and *USP48* (ubiquitin-specific peptidase 8 and 48) genes in adrenocorticotropic (ACTH) PitNETs, *PIK3AC* in various types of PitNEts, or in the *SF3B1* (Splicing Factor 3B subunit 1) gene in prolactin (PRL)-PitNETs [51].

The variant described in *SF3B1*, which causes aberrant splicing of estrogen-related receptor gamma and strengthens the binding of PIT1 (pituitary-specific positive transcription factor 1) and the estrogen-independent PRL (PRL-releasing peptide) transcription, is an example of a pathogenic mechanism involving splicing alterations.

Hence, besides the multistep model of tumorigenesis based on driver mutations, events that alter gene transcription or expression [52,53] may be worth investigating.

Different authors have drawn attention to another mechanism, splicing dysregulation, which is likely involved in tumor development [54], and preliminary studies published on PitNETs have unraveled the abnormal expression of oncogenic splicing variants [55,56,57,58,59,60].

Splicing alterations were also described in an ubiquitin-specific peptidase 29 (USP39)-mutant zebrafish that developed pituitary hyperplasia. The loss of USP39 led to an aberrant *RB1* mRNA splicing and expression decrease with consequences on downstream partners, i.e., transcription factors such as ef24 or p21 [61].

Furthermore, the spliceosome became an interesting therapeutic target due to pladienolide-B, a macrolide that inhibits the splicing factor SF3B1 with anti-tumorigenic effects [62,63].

A study of the expression of splicing components in 261 PitNETs of any type, and of pladienolide-B effect in cell lines, demonstrated a hallmark of dysregulation of spliceosome components in all analyzed PitNETs and the potential of the spliceosome as a therapeutic target [21].

## 5. Genomic Integrity

The identification of the pathways involved in NET development showed that, besides well-known pathways such as PTEN/mTOR, the most relevant or frequently altered genes have a role in histone modification and chromatin remodeling or telomere maintenance, e.g., *MEN1*, *DAXX*, *ATRX*, *SETD2*, *ARID1A*, and *MLL3,* with a variety of downstream effects on transcriptional regulation yet to be fully elucidated.

Han et al. [22] focused on nonsense mutations in *ARID1A* (AT-rich interactive domain-containing protein 1A) that encodes a member of the chromatin-remodeling complex SWI/SNF (switching defective/sucrose non-fermenting), which intervenes in transcriptional activation of genes in a heterochromatin state [64]. This gene is likely more relevant in tumorigenesis than *ARID1B* (AT-rich interactive domain-containing protein 1B) or *SMARC4/BRG1* (SWI/SNF-related, matrix-associated, actin-dependent regulator of chromatin, subfamily a, member 4, encoding for Brahma-related gene 1 protein), other components of the same complex, the latter just described as inactivated in NETs by rare chromosomal rearrangements [12]. Both *ARID1A* and *ARID1B* are known to be TSGs [65,66].

The authors found in non-functioning PanNETS that downregulating mutations in *ARID1A* were associated with higher proliferation index and aggressiveness. Its loss in mice led to inflammation and precancerous lesions, also confirming a role for *ARID1A* in pancreatic homeostasis [67,68].

It may be worth investigating which genes endure an impact from *ARID1A* alterations, indirectly or directly through their action on chromatin remodeling, a mechanism whose alteration has complex effects on transcriptional regulation, cellular homeostasis, and plasticity, as described in the subsequent paragraph.

## 6. Permissive Chromatin Landscape

The presence of mutations in genes involved in epigenetic regulation is not specific for NETs [69,70], but its high frequency in comparison to more common genetic alterations, such as those exploited for targeted therapies, is a peculiarity that guides the research perspective in this field.

Recently [23], Wasylyshen et al. explored, in mouse models, the role of *DAXX* and *ATRX*, the H3.3 histone variant chaperones, whose mutually exclusive mutations are found in about 43% of PanNETs [28]. The proteins encoded by *DAXX* and *ATRX* collaborate to form a heterodimeric complex that deposits the H3.3 histone variant into repetitive heterochromatin, including telomeres and retrotransposons [71]. Among the effects of its mutants, e.g., the lengthening of telomeres [72], *DAXX* plays a role in apoptosis and DNA damage response. *DAXX* loss is associated with dysregulation of heterochromatin [73], as also shown by whole-transcriptome analyses.

In addition, *DAXX,* together with *ATRX* and H3.3, silences transposable elements such as endogenous retroviral elements (ERVs) in mouse embryonic stem cells [74,75,76]. The human counterparts of these retroviral genomic elements integrated in the DNA are well known but the extent of their interference in tumorigenesis, immunity, or inflammation is not [77].

Indeed, as demonstrated through transcriptome and chromatin accessibility profiling by RNA-seq and ATAC-seq (Assay for Transposase-Accessible Chromatin using sequencing), *DAXX* loss leads to the de-repression of ERVs and altered expression of genes mediated by their long terminal repeat promoters.

A higher expression of *BGLAP3* (bone gamma-carboxyglutamate protein), an osteocalcin-related gene [78,79] that contains an intragenic ERV, was demonstrated by transcriptome analysis associated with *DAXX* and, to a lesser extent, *ATRX* loss.

These data suggest a permissive transcription state related to the histone chaperone function of both genes.

In addition, observation of Pdx/CreTg mice with different genotypes showed that *DAXX* loss is not tumorigenic *per se* in the pancreas. When combined with *MEN1* loss, it led to inflammatory stress and cystic degeneration of the exocrine pancreas, besides islet cell hyperplasia and PanNETs caused by *MEN1* loss alone, as expected (this effect is seen in human NETs also [12,28]).

In mice with caerulein-induced pancreatitis, *MEN1* loss potentiated the inflammatory response, while *DAXX* loss led to the up-regulation of a significant number of genes involved in the pancreas cell state, e.g., hepatic stellate cells’ pathway [80,81]. These studies, using global transcriptome analysis through RNA-seq, revealed cellular plasticity in pancreatic cells. The combination of *DAXX* and *MEN1* losses also impaired tissue recovery with the persistence of ductal metaplasia.

On one hand, *DAXX* and, therefore, H3.3 loss may alter the transcriptional state but also contribute to cell homeostasis alteration determined by *MEN1* loss [82], restraining cellular plasticity.

On the other hand, *MEN1*, known to regulate transcription factors, e.g., JunD, might contribute to an altered chromatin state. JunD is part of the AP-1 (Activator Protein 1) transcription factor complex and negative regulator of *RAS* [83]. Furthermore, *MEN1* activates the transcription of *CDKN1B* and *CDKN1C* (cyclin-dependent kinase inhibitor 1B and C) through the MLL (mixed lineage leukemia) protein and LEDGF/p75 (lens epithelium-derived growth factor) [84]. *CDKN1B* encodes p27Kip1, a CDK inhibitor that prevents cell cycle progression from G1 to S phase, thus acting as a tumor suppressor gene [85].

Interestingly, other transposable elements, e.g., long and short interspersed nuclear elements (LINE and SINE) were not deregulated.

Among the up-regulated genes, 13% of which are characterized by intragenic ERVs, two genes were found to be significantly up-regulated in *DAXX* mutant tumors: *FABP3* (Fatty-acid-binding protein 3) and Serine incorporator 2 (*SERINC2),* neighboring genes close to an ERV locus. The dysregulation of *SERINC2* and the nearby ERV have just been described in human lung adenocarcinoma also [86].

## 7. Aberrant Methylation

Another mechanism potentially implicated in PanNET tumorigenesis is the presence of aberrant methylation patterns.

Three genes in particular presented aberrant methylation in PanNETs: *MGMT* (O6-methylguanine-DNA methyltransferase) [87], *PDX1* (Pancreatic and Duodenal Homeobox 1) [88], and *CASP8* (caspase 8) [89,90].

A recent study [24] differentiated a cohort of sporadic PanNETs into three subgroups based on their methylation patterns and clinical and genomic features. The T1 groups included functional neoplasias with no variants in *ATRX*, *DAXX,* and *MEN1* as opposed to the T2 group, also characterized by larger size, longer telomeres, diffuse chromosomal LOH unrelated to the methylation levels, a high tumor mutational burden (especially in the mTOR pathway), and lower methylation of *MGMT*.

The T3 group showed *MEN1* variants, loss of chromosome 11, a lower frequency of invasion out of the site of origin, and hypermethylation of *PDX1* (also present in the T2 group and, to a lesser extent, in the T1 group; the T1 group included tumors from β-cells, while T2 and T3 included tumors from α-cells).

In summary, the description of methylation patterns can be another tool to help clinical stratification of NETs combined with other data or to establish cell of origin of a tumor.

Nevertheless, in the next paragraph we describe a study that deeply exploited the mechanism of the tumoral transformation of β-cells.

## 8. Cell Dedifferentiation

The absence of viable biomarkers of progression or metastatic potential, for the benefit of clinical staging or personalized treatments, proves that for NETs in particular a genomic perspective can be insufficient compared to a multiomics one.

A study based on this comprehensive approach revealed further insights on PanNETs’ tumorigenesis with a specific focus on the process of cell dedifferentiation to explain the evolution of well-differentiated islet tumors (IT) into metastasis-like primary (MLP) tumors [25].

The origin of PanNETs is debated, since their definition of islet cell tumors, while some authors proposed that they arise from stem cells of the ductal–acinar system. Previous studies suggested that MLP tumors arise from progenitor cells [91,92,93], while others hypothesized that dedifferentiation starts from cancer cells as a stage of malignant progression [94,95].

The authors demonstrated that β-cells can acquire a progenitor-like molecular phenotype due to differential expression of genes under the influence of a specific miRNA cluster, a chain of events that influences cellular plasticity and precedes malignant progression, hence temporally distinct from other known genetic determinants that drive cell proliferation.

On a side note, these authors also classified PanNETs into three subgroups on the basis of transcriptomic data, i.e., well-differentiated islet tumors (WD-IT), intermediate, and the poorly differentiated MLP tumors, overlapping the T1, T3, and T2 groups, respectively, of the previously cited study.

Molecular analysis of the tumor developed by the mouse model RT2 (RIP1-Tag2), characterized by the inactivation by the SV40 antigen of p53 and RB, allowed the identification of a subgroup of WD-IT and MLP tumors lacking the most frequent variants identified in human PanNETs [12] but with similar mRNA and miRNA transcriptome profiles [92,96], making it a valid model of comparison.

The analysis of mRNA and miRNA signatures revealed that MLP tumors highly expressed pancreatic progenitor-specific markers and genes with a role in maintaining stemlike features, embryonic development, and epithelial-to-mesenchymal transition (*Sox11*, *Sox6*, *Cited1*, *Id1*, *Zfp536*) or repressors of cell differentiation, while genes with a role in β-cell homeostasis were downregulated. The similarity between mRNA and miRNA profiles of MLP tumors and progenitor cells supported the hypothesis that MLP tumors derived from a process of dedifferentiation from WD-IT with a resurgence of a progenitor genes’ expression.

To identify the players leading this process, the authors focused on miRNAs, due to the similar profile between MLP tumors and progenitor cells and also to their role in cellular reprogramming [97,98], looking for miRNA peculiar to MLP tumors and progenitor cells, namely, miR-181c and miR-181d.

To assess a possible functional role, the miR-181cd cluster was conditionally overexpressed in βTC3 cell lines, which showed an IT-like phenotype. The activation of the progenitor-like program was evaluated by applying the MLP mRNA-signature onto the transcriptome profiles of the samples. Interestingly, seven days of miR-181cd overexpression in the βTC3 IT-like cancer cells resulted in the transition of these cells toward the MLP subtype, through the upregulation of genes involved in cell differentiation and the acquisition of a neuronal-like morphology.

Furthermore, specific algorithms, ARACNE [99,100] and VIPER [101], and datasets, Bio-miRTa 35, were adopted in order to identify the participating transcription factors and the genetic targets of miR-181cd.

The study deemed *Hmgb3* and *Meis2* as the most relevant genetic targets. Both genes encode homeobox proteins and, hence, are involved in differentiation events, and were just described in correlation with cancer aggressivity and stemness properties [102,103,104,105], the first resulting highly expressed in MLP tumor samples in consequence of the downregulation of *Meis2* and other IT markers.

In summary, the pathway formed by miR-182cd cluster, *Meis2,* and *HGMB3* represents a major player in a discrete step in PanNETs’ tumorigenesis that paves the way to the manifestation of a metastatic potential through the modulation of the cellular plasticity.

## 9. Conclusions

Extensive genomic studies are fundamental in the identification of the major genetic players of NEN tumorigenesis. Nevertheless, the biological effects of such alterations still require clarification. This is especially true when considering the wide range of functions carried out by genes such as *MEN1* or *DAXX* and their peculiar high frequency in NETs.

Notwithstanding the multistep model for tumorigenesis, the recent studies here reviewed and integrated demonstrated the relevance, notably for NETs, of the epigenetic and transcriptional levels and the need for models based on complex interactions and effects (e.g., on homeostasis and inflammation) other than a linear progression of events (see Figure 1).

Any data obtained from animal models require confirmation on human cells, due to possible interspecies’ differences in the anatomy of some organs or in the evolution and localization of transposable elements.

Zebrafish, other than mouse models, also seem very promising, due to the conservation of NET-related genes and neuroendocrine systems between zebrafish and humans, hence the possibility of studying, in an easily manageable, transplantable model, tumor progression, tumor microenvironment, or even therapeutic response, overcoming the heterogeneity of NETs [106,107].

Nevertheless, this compelling evidence, while definitively not exhaustive of any aspect of the development of such heterogenous tumors, proves that the identification of driver mutations is only one of the many steps necessary to encompass the chain of tumorigenic events and to identify novel prognostic biomarkers or therapeutic targets.

As new insights are mounting and NET tumorigenesis is elucidated, bench-to-bedside findings will become clinically useful, paving the way to new treatment strategies.

## Figures and Tables

**Table 1:** The table summarizes the main references, methods used, and results described for the mechanisms elucidated in the different sections of the review. Quality criteria for the selected articles were: being published within the last 2 years in high-level scientific journals (first two quartiles in the field of interest, https://www.scimagojr.com, accessed on 5 August 2021); scientific soundness; literature-based and/or prior studies from the same authors; methods’ description; specific predictors to existing model [17]; limitations due to the use of animal models reported [18]; and presence of citations, though being recently published.

Topic and Main Reference	Methods	Results
**Novel Tumor Suppressor Genes** [19]	*PHLDA3* Studies in Tissues from Human PanNETs, MIN6 Cells (β-Cells Derived), Transgenic *PHLDA3*-Deficient Mice.	Loss of *PHLDA3*, a p53 Target and Inhibitor of Akt, Disrupts the Balance of p53-PHLDA3-Akt Axis and Promotes NETs’ Tumorigenesis.
**Cooperative** **Tumorigenic** **Effects** [20]	Pairwise and Single Homozygous Deletions of *RB1*, *PTEN*, *MEN1*, p53 in Insulin II Gene Expressing Cells (Cre-LoxP System). Histopathology of the Pituitary and Pancreas in the Mice. Scoring of the Cooperative Role of the Aforementioned Genes in PitNETs and PanNETs.	In PitNETs, the Order of Relevance in Initiation and/or Progression was Established as *RB1*, *PTEN*, *MEN1,* and p53, while, as Expected, in Islet Tumorigenesis it was *MEN1*, *PTEN*, *RB1,* and Lastly p53.
**Dysregulation of Splicing Machinery in Pitnets** [21]	Analysis of the Expression Levels of Spliceosome Core Components by Dynamic qRT-PCR Microfluidic array in the Main PitNETs’ Subtypes. Scoring of mRNA Expression Levels. Evaluation of the mRNA and Protein Expression Levels of SF3B1 in Cell Lines Under Administration of Pladienolide-B.	Dysregulation of Splicing Machinery is a Unique Fingerprint in PitNETs and a Potential Therapeutic Target. Pladienolide-B Reduces Cell Proliferation and Hormone Secretion.
**Genomic Integrity** [22]	Exome Sequencing of Tissues from Sporadic PanNETs. The qRT-PCR and Immunohistochemistry to Evaluate mRNA Level and Protein Expression of *ARID1A*.	Loss of *ARID1A* Contributes to Tumorigenesis and Metastatic Behavior in Sporadic PanNETs.
**Permissive** **Chromatin** **Landscape** [23]	Conditional *DAXX* Allele in Mice. Whole-Transcriptome Analysis. Evidence of Dysregulation of Heterochromatin. Combination of *DAXX* Loss with *MEN1* Loss and/or Inflammatory Stress. Comprehensive Transcriptome and Chromatin Accessibility Profiling. Evidence of Dysregulation of ERVs, Gene Expression Changes, Altered Cell State, and Impaired Pancreas Recovery. RNA Sequencing on Human PanNETs and Evidence of Dysregulation of ERVs and Genes Downstream of *DAXX* Alteration.	*DAXX* Loss Leads to a Permissive Transcriptional State that, in Association with Environmental Stress and *Men1* Loss, Alters Gene Expression and Cell State. *DAXX* Loss-Associated Transcriptional Changes Dysregulate ERVs and Nearby Genes also in Human PanNETs.
**Aberrant Methylation** [24]	Genome-Wide Scan of DNA Methylation in PanNETs. Identification of Methylation Subgroups with Correlation to Clinical and Genomic Features.	Methylation Drives Tumorigenesis Together with Somatic LOH/Copy Number Changes and Contributes to the NETs’ Heterogeneity. Potential Role in Stratifying Prognosis and Supporting Therapeutic Choices for PanNETs.
**Cell Dedifferentiation** [25]	Profiling for mRNA and miRNa and Proteomic Analysis of Samples from Primary Tumors and Metastases from RT2 Genetically Engineered Mouse Models. Isolation of Two Clusters with Different Profiles and also Expression of Mature β–Cell or Progenitor Markers, i.e., Islet Tumors (IT) and Metastasis-Like Primary Tumors (MLP). Development of mRNA and miRNA Signature for the MLP Cluster. Identification of miRNAs Responsible for the Activation of the MLP Program in IT, i.e., miR-181c and miR-181d, Demonstrated by the Overexpression of this miRNA Cluster in the βTC3 Cell Line (IT-Like) with piggyBac Transposon System and the Application of the MLP mRNA Signature onto the Transcriptome Profiles of the Samples in Order to Evaluate the Activation of the Progenitor-like Program. Identification of the Transcription Factors Influenced by the mi-RNA Cluster and Regulating the Dedifferentiation from IT to MLP subtype.	Description of a Novel Mechanism that Modulates Cancer Cell Plasticity. MLP Tumors Arise from IT via Dedifferentiation and Acquisition of β-Cells’ Progenitor-Like Phenotype. The microRNA-181cd Cluster Induces the IT-to-MLP Transition by Suppressing Expression of *Meis2* and Consequent Upregulation of *Hmgb3*. IT-to-MLP Transition is a Discrete Step Preceding the Proliferation of Cancer Cells in Tumorigenesis.
